# Effect of comorbid pulmonary disease on the severity of COVID‐19: A systematic review and meta‐analysis

**DOI:** 10.1111/resp.14049

**Published:** 2021-05-06

**Authors:** Askin Gülsen, Inke R. König, Uta Jappe, Daniel Drömann

**Affiliations:** ^1^ Division of Clinical and Molecular Allergology Research Center Borstel, Leibniz Lung Center, Airway Research Center North (ARCN), Member of the German Center for Lung Research (DZL) Borstel Germany; ^2^ Interdisciplinary Allergy Outpatient Clinic, Department of Pneumology University of Luebeck Luebeck Germany; ^3^ Institute of Medical Biometry and Statistics Airway Research Center North (ARCN), Member of the German Center for Lung Research (DZL), University of Luebeck Luebeck Germany; ^4^ Department of Pneumology Airway Research Center North (ARCN), Member of the German Center for Lung Research (DZL), University of Luebeck Luebeck Germany

**Keywords:** coronavirus disease, COVID‐19, lung diseases, meta‐analysis, SARS‐CoV‐2, systematic review

## Abstract

Coronavirus disease 2019 (COVID‐19) caused by infection with severe acute respiratory syndrome coronavirus 2 was first detected in Wuhan, China, in late 2019 and continues to spread worldwide. Persistent questions remain about the relationship between the severity of COVID‐19 and comorbid diseases, as well as other chronic pulmonary conditions. In this systematic review and meta‐analysis, we aimed to examine in detail whether the underlying chronic obstructive pulmonary diseases (COPD), asthma and chronic respiratory diseases (CRDs) were associated with an increased risk of more severe COVID‐19. A comprehensive literature search was performed using five international search engines. In the initial search, 722 articles were identified. After eliminating duplicate records and further consideration of eligibility criteria, 53 studies with 658,073 patients were included in the final analysis. COPD was present in 5.2% (2191/42,373) of patients with severe COVID‐19 and in 1.4% (4203/306,151) of patients with non‐severe COVID‐19 (random‐effects model; OR = 2.58, 95% CI = 1.99–3.34, *Z* = 7.15, *p* < 0.001). CRD was present in 8.6% (3780/44,041) of patients with severe COVID‐19 and in 5.7% (16,057/280,447) of patients with non‐severe COVID‐19 (random‐effects model; OR = 2.14, 95% CI = 1.74–2.64, *Z* = 7.1, *p* < 0.001). Asthma was present in 2.3% (1873/81,319) of patients with severe COVID‐19 and in 2.2% (11,796/538,737) of patients with non‐severe COVID‐19 (random‐effects model; OR = 1.13, 95% CI = 0.79–1.60, *Z* = 0.66, *p* = 0.50). In conclusion, comorbid COPD and CRD were clearly associated with a higher severity of COVID‐19; however, no association between asthma and severe COVID‐19 was identified.

## Graphical abstract

**See related** Editorial

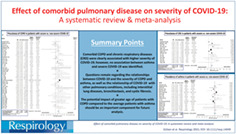

## INTRODUCTION

Coronavirus disease 2019 (COVID‐19) caused by infection with severe acute respiratory syndrome (SARS) coronavirus 2 (SARS‐CoV‐2) was first detected in Wuhan, China, in late 2019 and continues to spread worldwide. COVID‐19 can progress to debilitating pneumonia and SARS, particularly in elderly patients.^1^ As of 29 November, 61.8 million cases of COVID‐19, including 0.5 million new cases, have been reported worldwide with 1.4 million deaths.^2^ The current global recovery rate is 69.0%, and case fatality rates range from 1.3% to 9.8% depending on the country, with an average of 2.3% worldwide.^3^ Unlike past outbreaks of Middle East respiratory syndrome and SARS‐CoV, COVID‐19 has higher rates of human‐to‐human transmission and infectivity and a lower mortality rate.^4^, ^5^ Although there is not yet a clear consensus on treatment strategies for COVID‐19, various combinations of drugs, including anti‐malaria, anti‐viral and biological agents, have been used based on data obtained from short‐term experience with the disease.

It is of vital importance to identify patients at high risk for severe COVID‐19 as early as possible to interrupt the chain of infection by isolating them from the community. In a retrospective study investigating 85 fatal cases of COVID‐19, Du et al.^6^ reported that the average age of patients who died from COVID‐19 was 65.8 years, with a 72.9% male predominance and a high prevalence of comorbid diabetes, especially hypertension and coronary heart disease. Arrhythmia, acute respiratory distress syndrome, shock and respiratory failure were reported in 60.0%, 74.1%, 81.2% and 94.1% of patients, respectively. Therefore, the importance of identifying patients with COVID‐19 and comorbid disease has clearly been established.

Based on epidemiological data from patients with COVID‐19 in China, the prevalence of comorbid chronic respiratory disease (CRD) and comorbid chronic obstructive pulmonary disease (COPD) was 1.4% and 2.4%, respectively.^7^ The diseases specified as CRD were not defined in this study.^7^ Data on comorbid asthma remain unclear and are likely to be highly under‐reported. Various bacteria, including *Pseudomonas aeruginosa* and *Staphylococcus aureus*, and other respiratory viruses affect the mortality and morbidity associated with chronic pulmonary diseases by inducing disease exacerbations and causing community‐acquired pneumonia.^8^, ^9^ For this reason, it has long been recommended that COPD patients undergo routine influenza and pneumococcal vaccinations according to the Global Initiative for Chronic Obstructive Lung Disease (GOLD) guidelines and that patients with moderate‐to‐severe asthma undergo influenza vaccination according to the Global Initiative for Asthma (GINA) guidelines.^10^, ^11^ In addition, these patients are known to be more susceptible to respiratory infections due to the use of inhaled corticosteroids, bacterial colonization and microbiome changes in the lung, mucus overproduction, systemic inflammation, smoking history and nutritional disorders.^12^ It is not yet known how inhaled corticosteroids and biological drugs affect the course of COVID‐19. COVID‐19 seems to create different clinical scenarios, so the possible beneficial or harmful effects of these drugs should be clarified as soon as possible.

Although Chen et al.^13^ reported that comorbid COPD did not increase the severity of COVID‐19, a preliminary meta‐analysis including seven studies reported that patients with COPD did experience more severe COVID‐19.^14^ In addition, active smokers or COPD patients were reported in another study to have increased mortality rates.^15^ Besides, advising high‐risk patients with comorbid diseases to self‐isolate at home may have resulted in fewer hospitalizations in this group and, consequently, decreased representation in relevant studies. Therefore, persistent questions remain about the increased susceptibility of patients with comorbid COPD and other chronic pulmonary conditions to COVID‐19 infections in general and severe disease course in particular.^16^ Accordingly, in this systematic review and meta‐analysis, we aimed to compare the prevalence of COPD, asthma and undefined CRDs in severe and non‐severe COVID‐19 patients, and to examine their associated risk of a more severe course of COVID‐19.

## METHODS

Included articles were evaluated according to the Preferred Reporting Items for Systematic Reviews and Meta‐Analysis (PRISMA) statement.^17^ In addition, our meta‐analysis was recorded in the International Prospective Register of Systematic Reviews (PROSPERO) (https://www.crd.york.ac.uk/prospero; registration number: CRD42020179122).

### Literature search strategy

A comprehensive literature search was performed using five international search engines with the Cochrane Library, Google Scholar, PubMed, Scopus and Web of Science databases. The following search terms were used to identify relevant studies available prior to 20 October 2020: ‘COVID‐19’ OR ‘2019‐nCoV’ OR ‘SARS‐CoV‐2’ OR ‘novel coronavirus’ AND ‘COPD’ OR ‘asthma’ OR ‘respiratory disease’ AND ‘clinical characteristics’ OR ‘risk factor’. The details of our search strategy are shown in Table S1 in the Supporting Information. In the initial search, 327 articles were identified. On 19 October 2020, we re‐scanned the databases, and an additional 395 articles were identified.

### Inclusion and exclusion criteria

Articles identified through this search strategy were then evaluated with consideration of this study's eligibility criteria, namely: (1) comparative studies (non‐severe vs. severe disease); (2) epidemiological studies (cross‐sectional, observational, retrospective or prospective); and (3) diagnosis of COVID‐19 based on clinical, radiological and microbiological evaluations according to the World Health Organization criteria.

**FIGURE 1:**
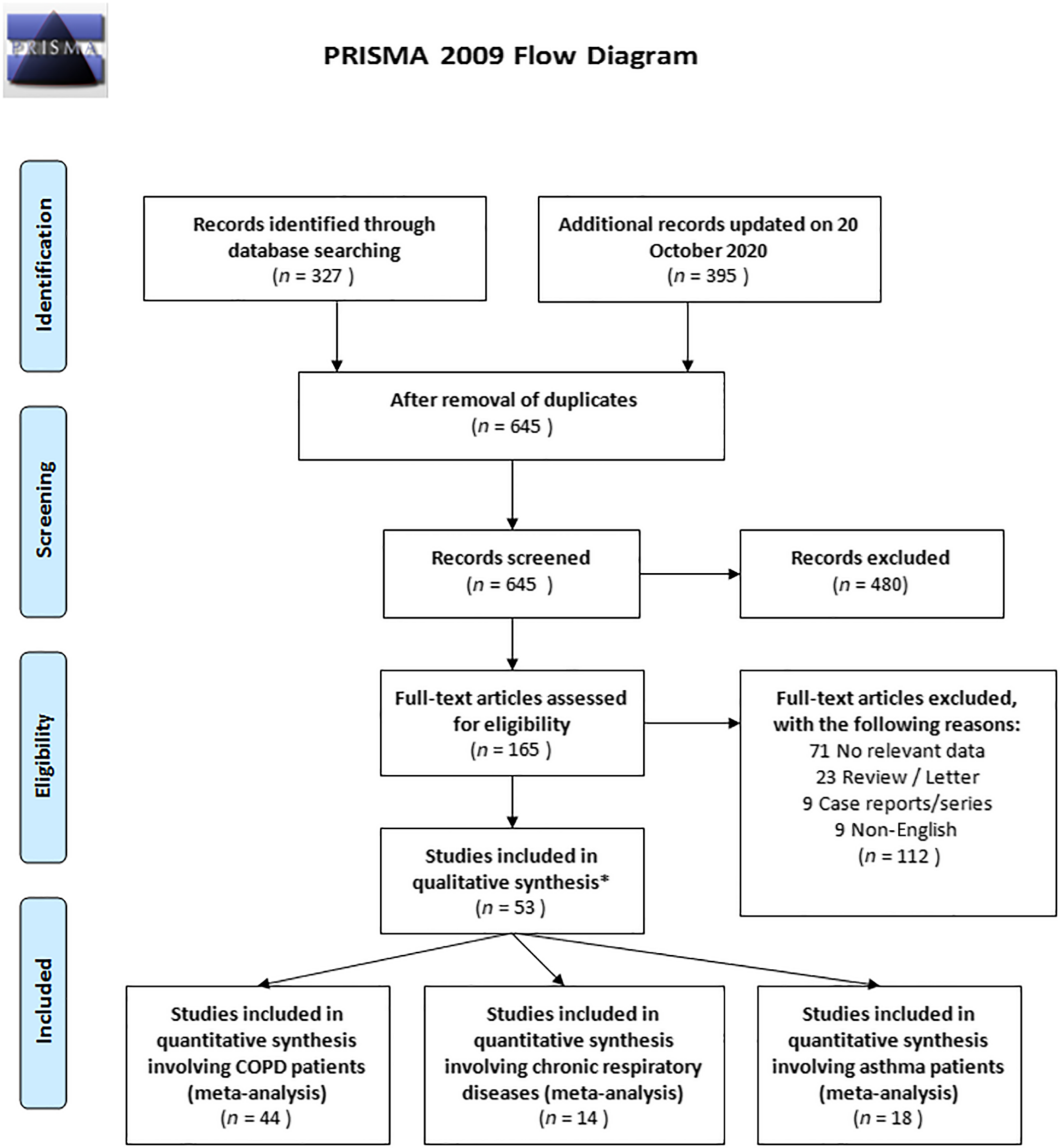
Study flow diagram of the inclusion criteria of included studies. * These studies provide data for more than one disease

Case reports/series, editorial letters, reviews, non‐English language articles and studies that did not compare non‐severe to severe COVID‐19 were excluded from this analysis. ‘Non‐severe’ cases were defined using various terminologies in different studies, including non‐severe, mild, common‐type, good outcome, recovered, no need for invasive mechanical ventilation (IMV), no need for intensive care unit (ICU) treatment, discharged and survivors. ‘Severe’ cases were also defined in different ways, including critical, poor outcome, severe, refractory to treatment, need for IMV, need for ICU and non‐survivors. The study flow diagram is shown in Figure 1.

### Data extraction

These articles were scanned in detail according to the inclusion criteria, and the resultant studies were subjected to quality evaluations and data extraction by two independent reviewers (AG and DD). The obtained data (first author, publication year, city and country of publication, mean age and disease prevalence according to the severity of COVID‐19) were recorded. Any disputes over the included studies were resolved by a third investigator (UJ).

### Quality and risk of bias assessments

The methodological index for nonrandomized studies (MINORS) tool was used to assess the quality and risk of bias.^18^ This tool includes the evaluation of eight sections, as follows: (1) a clearly stated aim; (2) inclusion of consecutive patients; (3) prospective collection of data; (4) endpoints appropriate to the aim of the study; (5) unbiased assessment of the study endpoint; (6) follow‐up period appropriate to the aim of the study; (7) loss to follow‐up less than 5%; and (8) 2 points (reported and adequate). According to this evaluation, studies were categorized as: (1) very low quality (0–4 points), (2) low quality (5–8 points), (3) medium quality (9–12 points) and (4) high quality (13–16 points).

### Data synthesis and statistical analysis

Statistical analysis and meta‐analysis were performed using OpenMeta Analyst software version 10.10 (https://www.cebm.brown.edu/open_meta) and StatsDirect version 3.2.10 (StatsDirect Ltd, Cambridge, UK). The prevalence of pulmonary diseases in patients with non‐severe and severe COVID‐19 was collected in a meta‐analysis pool, and ORs and 95% CIs were calculated. Heterogeneity among studies in the pool were evaluated using Cochran's *Q* test and Higgins' *I* ^2^ test. Homogeneity was accepted if a *p*‐value of >0.1 and an *I* ^2^ of <50% were obtained, and a fixed‐effect model was used. However, if the *I* ^2^ was ≥50%, a random‐effects model was used. Forest plots were then used to show the prevalence of asthma, COPD and CRD in patients with non‐severe and severe COVID‐19 in pooled studies. Egger's test and funnel plots were used to assess publication bias. Two‐sided *p‐*values of <0.05 were considered to indicate significance, except for evaluations using the *I* ^2^ heterogeneity test.

## RESULTS

### Study selection procedures

After the initial search in October 2020, 722 relevant articles from international databases were identified. After eliminating duplicate records, 645 articles remained in the pool, and, after abstract and title review, 165 articles remained in the pool. After consideration of the eligibility criteria, a further 112 studies were eliminated. Thus, 53 studies^13^, ^19^, ^20^, ^21^, ^22^, ^23^, ^24^, ^25^, ^26^, ^27^, ^28^, ^29^, ^30^, ^31^, ^32^, ^33^, ^34^, ^35^, ^36^, ^37^, ^38^, ^39^, ^40^, ^41^, ^42^, ^43^, ^44^, ^45^, ^46^, ^47^, ^48^, ^49^, ^50^, ^51^, ^52^, ^53^, ^54^, ^55^, ^56^, ^57^, ^58^, ^59^, ^60^, ^61^, ^62^, ^63^, ^64^, ^65^, ^66^, ^67^, ^68^, ^69^, ^70^ remained, which included data related to COPD (*n* = 44), CRD (*n* = 14) and asthma (*n* = 18). The general characteristics, locations, comparisons and prevalence data of these studies are presented in Table 1.

**TABLE 1:** Summary of studies included in the meta‐analysis

Study	Location	Design	*n*	Comparison	Age (years)	Asthma, *n* (%)	COPD, *n* (%)	CRD, *n* (%)
Almazeedi^19^	Kuwait	R, SC	1096	Non‐ICU (*n* = 1054) versus ICU (*n* = 42)	41.0	43 (3.9)	5 (0.5)	—
Argenziano^20^	New York, USA	R, MC	1000	Non‐ICU (*n* = 764) versus ICU (*n* = 236)	63.0	113 (11.3)	66 (6.6)	223 (22.3)
Auld^21^	Atlanta, USA	P, SC	217	Survivors (*n* = 147) versus non‐survivors (*n* = 62)	64.0	19 (8.8)	21 (9.7)	—
Berenguer^22^	Spain	R, MC	4035	Survivors (*n* = 2904) versus non‐survivors (*n* = 1131)	70.0	299 (7.5)	—	715 (17.9)
Buckner^23^	Seattle, USA	R, MC	105	Non‐severe (*n* = 54) versus severe (*n* = 51)	69	10 (10.0)	11 (10.0)	—
Cai^24^	Shenzhen, China	P, SC	383	Non‐severe (*n* = 292) versus severe (*n* = 91)	—	—	32 (8.3)	—
Cao^25^	Wuhan, China	R, SC	102	Survivors (*n* = 85) versus non‐survivors (*n* = 17)	54.0	—	—	10 (9.8)
Caratozzolo^26^	Italy	P, SC	848	Survivors (*n* = 807) versus non‐survivors (*n* = 41)	79.7^a^	—	73 (8.6)	—
CDC COVID‐19 Response Team^27^	—	R, MC	7162	Non‐ICU (*n* = 6180) versus ICU (*n* = 457)	—	—	—	656 (9.2)
Chen^13^	Zhejiang, China	R, SC	145	Non‐severe (*n* = 102) versus severe (*n* = 43)	47.5	—	6 (4.1)	—
Deng ^28^	Wuhan, China	R, MC	225	Survivors (116) versus non‐survivors (*n* = 109)	54.0	—	—	25 (11.1)
European Centre for Disease Prevention and Control, Week 43^29^	European Union	R, MC	263,654	Non‐severe (*n* = 224,506) versus severe (*n* = 39,148)	—	3625 (1.4)	—	11,601 (4.4)
Feng ^30^	Wuhan, China	P, SC	114	Good outcome (*n* = 94) versus poor (*n* = 20)	63.9^a^	—	11 (9.6)	—
Feng ^31^	Wuhan, China	R, MC	476	Non‐severe (*n* = 352) versus severe (*n* = 124)	—	—	22 (4.6)	—
Gao ^32^	Fuyang, China	R, SC	43	Mild (*n* = 28) versus severe (*n* = 15)	43.7^a^	—	3 (6.9)	—
Giorgi Rossi ^33^	Italy	P, SC	2653	Hospitalized (*n* = 1075) versus death (*n* = 217)	63.2^a^	—	128 (5.4)	—
Goyal ^34^	New York, USA	R, MC	393	Non‐IMV (*n* = 263) versus IMV (*n* = 130)	62.2	49 (12.5)	20 (5.1)	—
Grein ^35^	International	P, MC	53	Non‐IMV (*n* = 19) versus IMV (*n* = 34)	67.0	6 (11.0)	—	—
Guan ^36^	Outside Hubei, China	R, MC	1099	Non‐severe (*n* = 926) versus severe (*n* = 173)	47.0	—	12 (1.1)	—
Gupta ^37^	USA	P, MC	2215	Survivors (*n* = 1431) versus non‐survivors (*n* = 784)	60.5	258 (11.6)	178 (7.8)	531 (24.0)
Güner ^38^	Turkey	R, SC	222	Mild (*n* = 172) versus critical (*n* = 50)	50.6^a^	—	12 (5.4)	—
Harrison ^39^	TriNetX Study, USA	R, MC	31,461	Survivors (30,165) versus non‐survivors (*n* = 1296)	50.0	—	—	5513 (17.5)
He ^40^	Wuhan, China	R, SC	336	Survivors (*n* = 203) versus non‐survivors (*n* = 133)	65.0	—	28 (8.3)	—
Hu ^41^	Wuhan, China	R, SC	323	Non‐severe (*n* = 151) versus severe (*n* = 172)	61.0	—	6 (1.9)	29 (9.0)
Huang ^42^	Wuhan, China	P, SC	41	Non‐ICU (*n* = 28) versus ICU (*n* = 13)	49.0	—	1 (2.4)	—
Israelsen ^43^	Denmark	R, SC	175	Non‐ICU (*n* = 148) versus ICU (*n* = 27)	71.0	20 (11.4)	11 (6.3)	—
Javanian ^44^	Iran	R, SC	100	Survivors (*n* = 81) versus non‐survivors (*n* = 19)	60.1^a^	—	12 (12.0)	—
Lagi ^45^	Italy	R, SC	84	Non‐ICU (*n* = 68) versus ICU (*n* = 16)	62.0	—	5 (5.9)	—
Liu ^46^	Wuhan, China	P, MC	78	Improvement (*n* = 67) versus progression (*n* = 11)	38.0	—	2 (2.5)	—
Li ^47^	Wuhan, China	R, SC	548	Non‐severe (*n* = 279) versus severe (*n* = 269)	60.0	5 (0.9)	17 (3.1)	—
Li ^48^	Wuhan, China	R, SC	25	Non‐severe (*n* = 16) versus severe (*n* = 9)	—	—	5 (20.0)	—
Mo ^49^	Wuhan, China	R, SC	155	General (*n* = 70) versus refractory (*n* = 85)	54.0	—	5 (3.2)	—
Parra‐Bracamonte ^50^	Mexico	R, MC	331,298	Survivors (*n* = 292,988) versus non‐survivors (*n* = 38,310)	44.0	8983 (2.7)	5458 (1.6)	—
Paranjpe ^51^	New York, USA	R, MC	2199	Discharged (*n* = 768) versus mortality (*n* = 310)	65.0	180 (8.2)	113 (5.1)	—
Qi ^52^	Chongqing, China	R, MC	267	Non‐severe (*n* = 217) versus severe (*n* = 50)	48.0	—	—	25 (9.4)
Salacup ^53^	Philadelphia	R, SC	242	Survivors (*n* = 190) versus non‐survivors (*n* = 52)	66.0	18 (7.0)	30 (12.0)	—
Shi ^54^	Wuhan, China	R, SC	671	Survivors (*n* = 609) versus non‐survivors (*n* = 62)	63.0	—	23 (3.4)	—
Tomlins ^55^	UK	R, SC	95	Survivors (*n* = 75) versus non‐survivors (*n* = 20)	75.0	21 (22.0)	10 (11.0)	—
Wan ^56^	Chongqing, China	R, SC	135	Mild (*n* = 95) versus severe (*n* = 40)	47.0	—	4 (2.9)	5 (3.7)
Wang ^57^	Wuhan, China	R, SC	138	Non‐ICU (*n* = 102) versus ICU (*n* = 36)	56.0	—	4 (2.9)	—
Wang ^58^	Wuhan, China	R, SC	339	Survivors (*n* = 274) versus non‐survivors (*n* = 65)	71.0	—	21 (6.2)	—
Wang ^59^	Wuhan, China	R, SC	69	SpO_2_ ≥ 90% (*n* = 55) versus SpO_2_ < 90% (*n* = 14)	42.0	2 (2.9)	4 (5.7)	—
Wu ^60^	Yancheng, Fuyang, Wuxi, China	R, MC	280	Mild (*n* = 197) versus severe (*n* = 83)	43.1^a^	—	1 (0.3)	6 (2.1)
Yan ^61^	Wuhan, China	R, MC	1004	Survivors (*n* = 964) versus non‐survivors (*n* = 40)	—	—	8 (0.8)	147 (14.6)
Yang ^62^	Chongqing, China	R, SC	133	Mild (*n* = 65) versus severe (*n* = 68)	—	—	4 (3.0)	—
Yang ^63^	Wuhan, China	R, SC	52	Survivors (*n* = 20) versus non‐survivors (*n* = 32)	51.9	—	—	4 (7.7)
Zhang ^64^	Wuhan, China	R, SC	140	Non‐severe (*n* = 82) versus severe (*n* = 58)	57.0	0 (0)	2 (1.4)	—
Zhang ^65^	Wuhan, China	R, SC	221	Non‐severe (*n* = 166) versus severe (*n* = 55)	55.0	—	6 (2.7)	—
Zhang ^66^	Wuhan, China	R, SC	111	Discharge (*n* = 93) versus deterioration (*n* = 18)	38.0	—	3 (2.7)	—
Zhang ^67^	Wuhan, China	R, SC	120	Common type (*n* = 90) versus severe (*n* = 30)	45.4^a^	—	4 (3.0)	—
Zhao ^68^	New York, USA	R, SC	641	Non‐ICU (*n* = 398) versus ICU (*n* = 195)	60.0	41 (6.4)	36 (5.6)	—
Zheng ^69^	Changsha, China	R, SC	161	Non‐severe (*n* = 131) versus severe (*n* = 30)	45.0	—	6 (3.7)	—
Zhou ^70^	Wuhan, China	R, MC	191	Survivors (*n* = 137) versus non‐survivors (*n* = 54)	56.0	—	6 (3.1)	—
Overall			658,073			0%–22.0%	0.3%–20.0%	2.1%–24.0%

*Note*: Age‐related data were given in median years.

Abbreviations: COPD, chronic obstructive pulmonary disease; COVID‐19, coronavirus disease 2019; CRD, chronic respiratory diseases (undefined lung diseases); ICU, intensive care unit; IMV, invasive mechanical ventilation; MC, multicentre; *n*, participants; P, prospective; R, retrospective; SC, single centre; SpO_2_, peripheral capillary oxygen saturation.

^a^ Mean values.

### Quality assessment and risk of bias summary

The average score of the included articles according to the MINORS assessment was 10.9 points (range: 6–14). A total of eight studies were prospective, and the remaining 45 studies were retrospective. There were seven low‐quality, 35 medium‐quality and 10 high‐quality studies. One study^29^ was not evaluated because it represented only a weekly report. The quality and bias risk assessments are summarized in Table 2.

**TABLE 2:** Bias risk assessment

Study	❶	❷	❸	❹	❺	❻	❼	❽	Score
Almazeedi ^19^	2	0	1	2	2	2	2	0	11
Argenziano ^20^	1	2	1	2	0	2	2	0	10
Auld ^21^	2	2	1	2	0	2	2	0	12
Berenguer ^22^	2	2	1	2	1	2	2	0	12
Buckner ^23^	1	2	1	2	0	2	2	0	10
Cai ^24^	2	2	2	2	0	2	2	0	12
Cao ^25^	2	2	0	2	0	2	2	0	10
Caratozzolo ^26^	2	2	2	2	1	1	0	0	10
CDC COVID‐19 Response Team^27^	2	1	1	2	1	2	1	0	10
Chen ^13^	2	2	1	2	0	2	2	0	11
Deng^28^	2	0	1	0	0	2	2	0	7
European Centre for Disease Prevention and Control, Week 43^29^	—	—	—	—	—	—	—	—	—
Feng ^30^	2	2	2	2	2	2	2	0	14
Feng ^31^	1	2	1	2	0	2	2	0	10
Gao ^32^	2	2	1	2	0	2	2	0	11
Giorgi Rossi ^33^	2	2	1	2	0	2	1	0	10
Goyal ^34^	2	2	1	2	2	2	2	0	13
Grein ^35^	0	2	2	2	0	2	1	1	10
Guan ^36^	2	1	1	2	2	2	1	0	11
Gupta ^37^	2	2	2	2	1	2	2	0	13
Güner ^38^	2	1	0	2	0	1	2	0	8
Harrison ^39^	2	1	1	2	1	2	2	0	11
He ^40^	2	2	1	2	0	2	2	1	12
Hu ^41^	2	1	0	2	1	2	2	0	10
Huang ^42^	2	2	2	2	2	2	2	0	14
Israelsen ^43^	2	2	1	2	0	1	2	0	10
Javanian ^44^	1	2	1	2	0	0	2	0	8
Lagi ^45^	2	2	2	2	0	2	2	0	12
Liu ^46^	2	0	0	2	0	2	2	0	8
Li ^47^	2	2	1	2	2	2	2	0	13
Li ^48^	2	0	1	2	0	2	2	0	9
Mo ^49^	1	2	1	0	0	0	2	0	6
Parra‐Bracamonte ^50^	2	2	1	2	0	2	2	0	11
Paranjpe ^51^	2	2	1	2	2	1	2	0	12
Qi ^52^	2	2	1	2	0	2	2	0	11
Salacup ^53^	2	1	1	2	1	2	0	0	9
Shi ^54^	2	1	1	2	2	2	2	0	12
Tomlins ^55^	1	2	0	2	0	1	2	0	8
Wan ^56^	2	2	0	2	0	1	2	0	9
Wang ^57^	2	2	1	2	2	2	2	0	13
Wang ^58^	2	2	1	2	0	2	2	0	11
Wang ^59^	2	2	0	2	2	1	2	0	11
Wu ^60^	2	0	1	0	0	1	2	0	6
Yan ^61^	2	1	0	2	0	2	2	0	9
Yang ^62^	2	0	1	2	0	2	2	0	9
Yang ^63^	2	2	1	2	2	2	2	0	13
Zhang ^64^	2	0	1	2	2	2	0	0	9
Zhang ^65^	2	0	1	2	2	2	2	0	11
Zhang ^66^	2	2	1	2	2	2	2	0	13
Zhang ^67^	2	2	1	2	2	2	2	0	13
Zhao ^68^	2	1	0	2	2	2	2	0	11
Zheng ^69^	2	2	1	2	0	2	2	0	11
Zhou ^70^	2	2	1	2	2	2	2	0	13

### Primary outcome

From these 53 studies,^13^, ^19^, ^20^, ^21^, ^22^, ^23^, ^24^, ^25^, ^26^, ^27^, ^28^, ^29^, ^30^, ^31^, ^32^, ^33^, ^34^, ^35^, ^36^, ^37^, ^38^, ^39^, ^40^, ^41^, ^42^, ^43^, ^44^, ^45^, ^46^, ^47^, ^48^, ^49^, ^50^, ^51^, ^52^, ^53^, ^54^, ^55^, ^56^, ^57^, ^58^, ^59^, ^60^, ^61^, ^62^, ^63^, ^64^, ^65^, ^66^, ^67^, ^68^, ^69^, ^70^ data from 658,073 patients were included in the pool, with average ages ranging from 38.0 to 79.7 years. In these studies, the average prevalence of COPD was 0.9% (range: 0.3%–20.0%, *n* = 6435), that of CRD was 2.9% (range: 2.1%–24.0%, *n* = 19,490) and that of asthma was 2.0% (range: 0%–22.0%, *n* = 13,692). The distributions of patients with COPD, CRD and asthma according to the severity of the COVID‐19 are presented in Tables S2, S3 and S4, respectively, in the Supporting Information.

### Chronic obstructive pulmonary disease and COVID‐19

**FIGURE 2:**
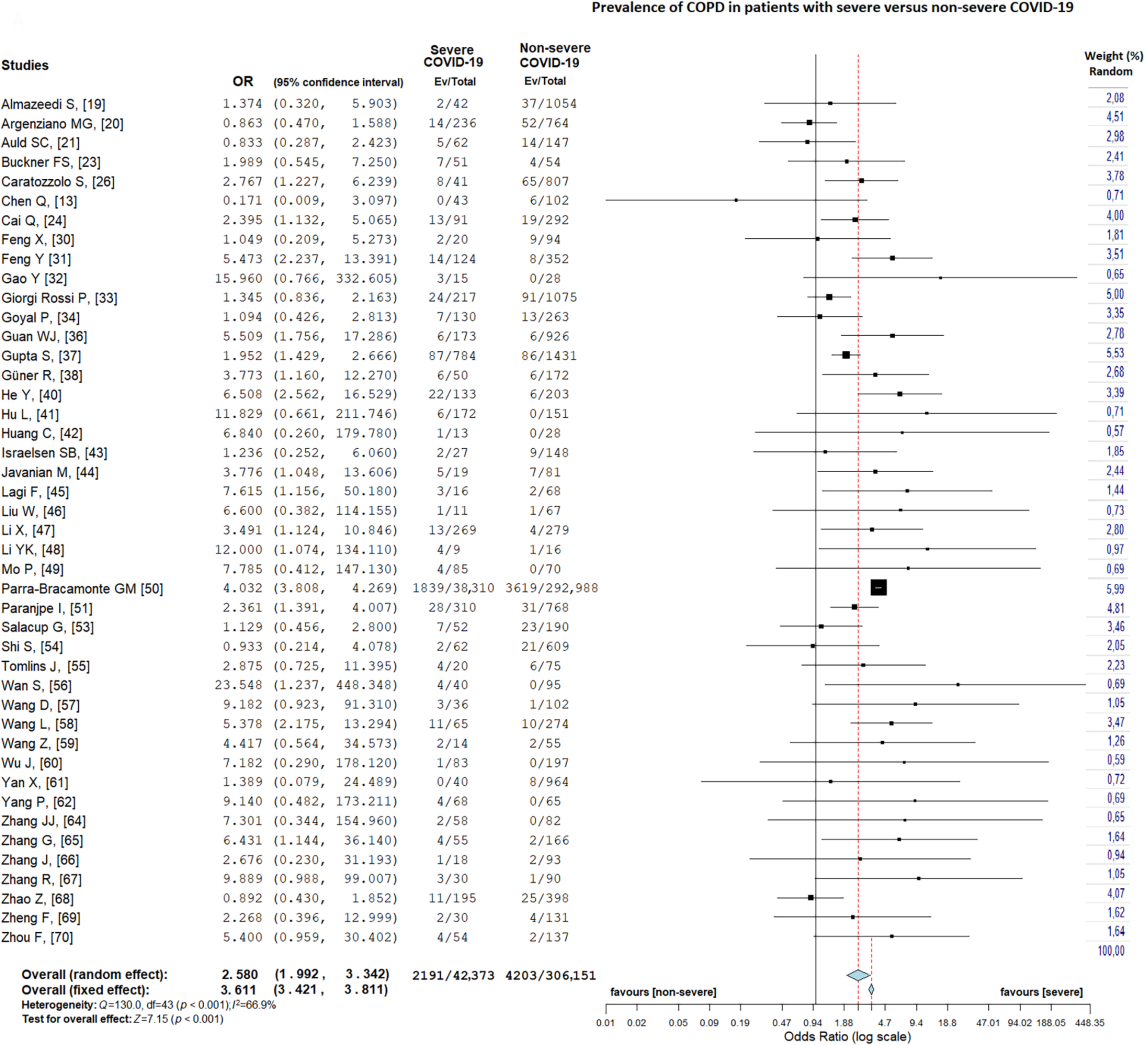
Prevalence of chronic obstructive pulmonary disease in patients with severe versus non‐severe coronavirus disease 2019 (COVID‐19)

COPD was present in 5.2% (2191/42,373) of patients with severe COVID‐19 and in 1.4% (4203/306,151) of patients with non‐severe COVID‐19 (random‐effects model; OR = 2.58, 95% CI = 1.99–3.34, *Z* = 7.15, *p* < 0.001; Figure 2), with substantial heterogeneity (*I* ^2^ = 66.9%, *p* < 0.001). Egger's test showed publication bias (*t* = −0.63, *p* = 0.02), but the funnel plot did not confirm this bias (Figure S1A in the Supporting Information). An additional sensitivity analysis was performed, as one study^50^ was considered to be the major cause of heterogeneity due to large number of patients. After removing this study,^50^ similar results (random‐effects model; OR = 2.42, 95% CI = 1.91–3.09, *Z* = 7.20, *p* = 0.005) were obtained, with low heterogeneity (*I* ^2^ = 39.7%, *p* < 0.001). The funnel plot distribution is presented in Figure S1B in the Supporting Information.

**FIGURE 3:**
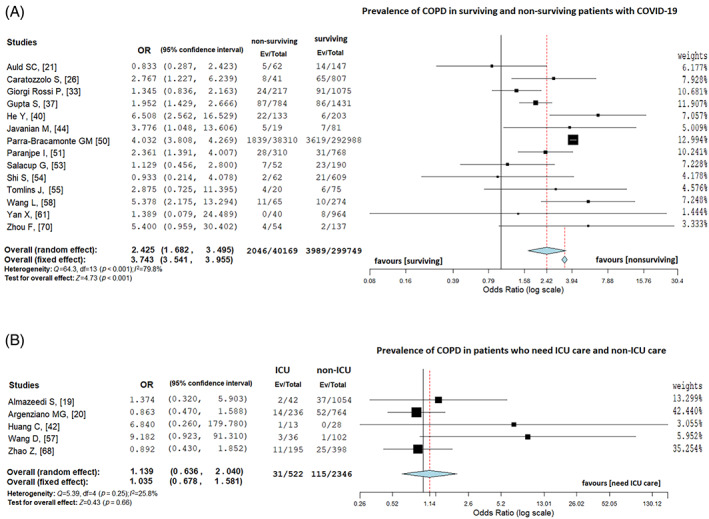
(A) Prevalence of chronic obstructive pulmonary disease (COPD) in surviving and non‐surviving patients with coronavirus disease 2019 (COVID‐19). (B) Prevalence of COPD in patients with COVID‐19 who needed intensive care unit (ICU) and non‐ICU treatment

In a subgroup analysis of pooled data, the prevalence of COPD was 1.3% (3989/299,749) in survivors and 5.1% (2046/40,169) in non‐survivors (random‐effects model; OR = 2.42, 95% CI = 1.68–3.50, *Z* = 4.73, *p* < 0.001; Figure 3A), with considerable heterogeneity (*I* ^2^ = 79.9%, *p* < 0.001). Egger's test shows publication bias (*t* = −1.44, *p* = 0.02) and the funnel plot supports this finding (Figure S1C in the Supporting Information). After removing Parra‐Bracamonte et al.,^50^ similar results (random‐effects model; OR = 2.20, 95% CI = 1.60–3.03, *Z* = 4.786, *p* < 0.001) were obtained with moderate heterogeneity (*I* ^2^ = 45.5%, *p* = 0.04). Egger's test provided no publication bias (*t* = 0.41, *p* = 0.57), and the funnel plot showed symmetrical distribution (Figure S1D in the Supporting Information).

In addition, the prevalence of COPD was 5.9% (31/522) in patients who needed ICU care and 4.9% (115/2346) in patients who did not need ICU care (random‐effect model; OR = 1.13, 95% CI = 0.64–2.04, *Z* = 0.43, *p* = 0.66; Figure 3B), with low heterogeneity (*I* ^2^ = 25.9%, *p* = 0.25). As the number of studies in this analysis was less than 10, we did not apply publication bias analysis.

### Chronic respiratory disease and COVID‐19

**FIGURE 4:**
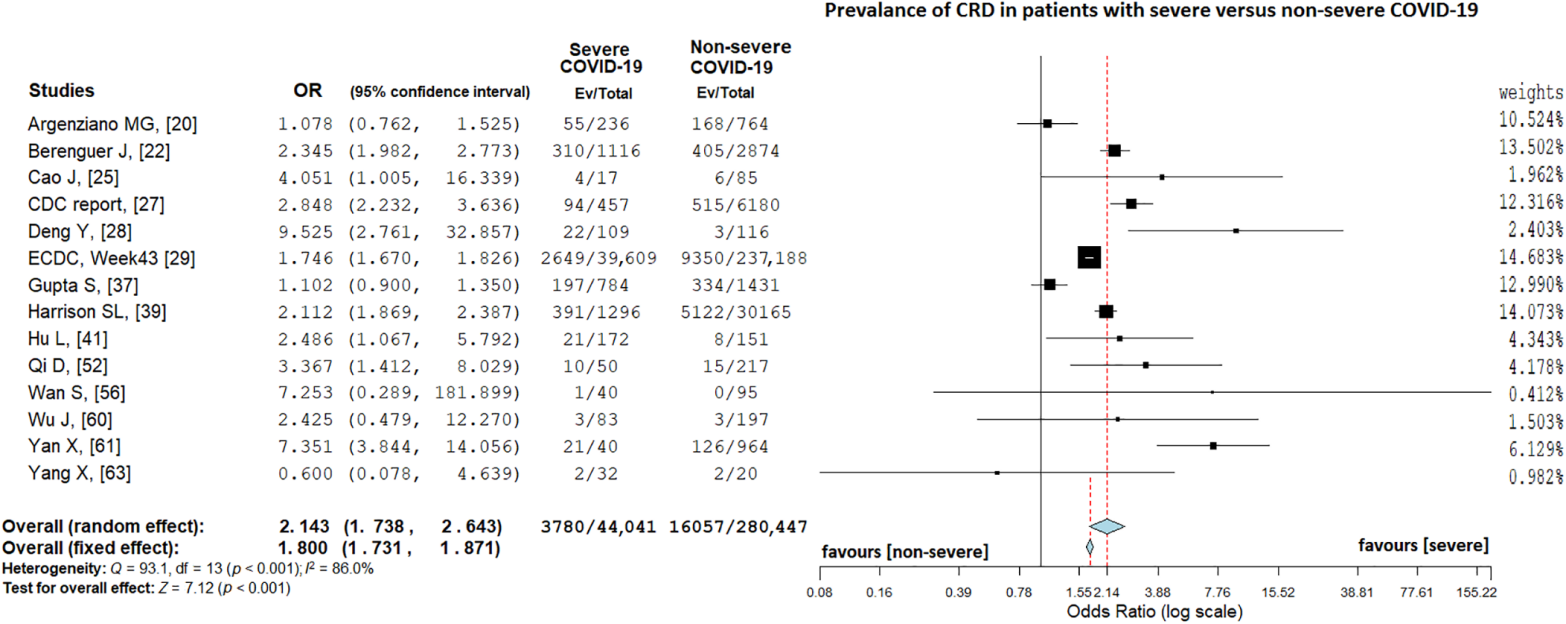
Prevalence of chronic respiratory disease in patients with severe versus non‐severe coronavirus disease 2019 (COVID‐19)

Fourteen articles^20^, ^22^, ^25^, ^27^, ^28^, ^29^, ^37^, ^39^, ^41^, ^52^, ^56^, ^60^, ^61^, ^63^ included data related to CRD. Because of considerable heterogeneity (*I* ^2^ = 86.0%, *p* < 0.001), the random‐effects model was used. CRD was present in 8.6% (3780/44,041) of patients with severe COVID‐19 and in 5.7% (16,057/280,447) of patients with non‐severe COVID‐19 (random‐effects model; OR = 2.14, 95% CI = 1.74–2.64, *Z* = 7.1, *p* < 0.001; Figure 4). As a result of sensitivity and leave‐one‐out analysis, no change in heterogeneity was obtained. The Egger's test was not significant (*t* = 1.09, *p* = 0.22) and provided no publication bias. The funnel plot distribution is presented in Figure S1E in the Supporting Information.

### Asthma and COVID‐19

**FIGURE 5:**
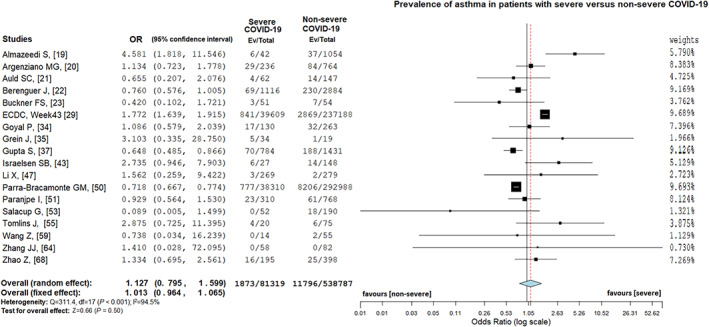
Prevalence of asthma in patients with severe versus non‐severe coronavirus disease 2019 (COVID‐19)

Eighteen articles^19^, ^20^, ^21^, ^22^, ^23^, ^29^, ^34^, ^35^, ^37^, ^43^, ^47^, ^50^, ^51^, ^53^, ^55^, ^59^, ^64^, ^68^ presented data on patients with asthma and COVID‐19, and the random‐effects model was used because of considerable heterogeneity (*I* ^2^ = 94.5%, *p* < 0.001). Asthma was present in 2.3% (1873/81,319) of patients with severe COVID‐19 and in 2.2% (11,796/538,737) of patients with non‐severe COVID‐19 (random‐effects model; OR = 1.13, 95% CI = 0.79–1.60, *Z* = 0.66, *p* = 0.50; Figure 5). The funnel plot was distributed symmetrically (Figure S1F in the Supporting Information), and Egger's test did not prove a publication bias (*t* = −0.06, *p* = 0.96). However, sensitivity and leave‐out analysis were performed, and two studies,^29^, ^50^ with large sample size which were found to be the cause of heterogeneity, were excluded. The result was similar to the first analysis (random‐effects model; OR = 1.08, 95% CI = 0.80–1.46, *Z* = 0.66, *p* = 0.50) with moderate heterogeneity (*I* ^2^ = 53.0%, *p* = 0.60). The Egger's test was not significant (*t* = 0.91, *p* = 0.16) and funnel plot (Figure S1G in the Supporting Information) did not show a publication bias.

## DISCUSSION

This comprehensive meta‐analysis examined the relationship between common chronic lung diseases, including COPD and asthma, and the severity of COVID‐19. In addition, our analysis included patients who were identified to have chronic respiratory or lung diseases, although these diseases were not fully defined. In summary, we observed that the severity of COVID‐19 was higher in patients with comorbid COPD and CRD, whereas COVID‐19 was not more severe in patients with comorbid asthma.

Thus far, the published research has not clearly identified the relationship between COPD and COVID‐19 severity. Although some initial studies reported that patients with COPD have a higher risk for a more severe COVID‐19 course,^24^, ^26^, ^31^, ^32^, ^38^, ^40^, ^41^, ^44^, ^45^, ^48^, ^57^, ^58^, ^62^, ^65^, ^67^, ^70^ other studies have reported conflicting results,^13^, ^30^, ^42^, ^43^, ^46^, ^47^, ^49^, ^53^, ^54^, ^55^, ^59^, ^61^, ^64^, ^66^, ^68^, ^69^ highlighting the need for further analyses. Data from five studies^19^, ^20^, ^42^, ^57^, ^68^ found no relationship between COPD and the need for ICU care.

A recently published study included 1590 patients with COVID‐19 and examined the prevalence of comorbid diseases in this population.^71^ In that study, the prevalence of COPD was 24 of 1590 (1.5%), with ICU admission required in seven of 24 (29.2%) patients with COPD, IMV required in five of 24 (20.8%) and death occurring in five of 24 (25%) patients. In non‐COPD patients, these rates were only 92 of 1566 (5.9%), 45 of 1566 (2.9%) and 44 of 1566 (2.8%), respectively.^71^ In the study of Guan et al.,^71^ COPD prevalence was reported as 1.5% and increased the hazard ratio (HR) for ICU, IMV and death (HR = 2.681, 95% CI = 1.42–5.05, *p* = 0.002). These findings are similar to our study (COPD prevalence, 0.9%; OR = 2.58, 95% CI = 1.99–3.34, *p* < 0.001).

Considering the chronic inflammatory state and low respiratory capacity of patients with COPD, it is not surprising that these patients are more likely to experience a more severe or even critical COVID‐19 course. No published studies have, however, grouped patients according to their COPD severity, which remains a topic for future research.

Some included studies used more general terms, such as CRD or ‘lung diseases’, to describe comorbid respiratory conditions.^20^, ^22^, ^25^, ^27^, ^28^, ^29^, ^37^, ^39^, ^41^, ^52^, ^56^, ^60^, ^61^, ^63^ While five of these studies^22^, ^28^, ^41^, ^52^, ^61^ identified a significant relationship between CRD and severe COVID‐19, one study^25^ did not identify any significant relationship (7.1% in mild cases and 23.5% in severe cases, *p* = 0.10). Other similar studies have provided epidemiological data without any statistical analysis.^20^, ^27^, ^29^, ^37^, ^39^, ^56^, ^60^, ^63^ Based on our meta‐analysis, patients with pre‐existing CRD were more likely to experience severe COVID‐19; however, the implications of this finding remain unclear because these diseases were not more specifically defined. It is interesting to note that the prevalence of CRD in COVID‐19 patients seems to be quite lower than that in the general population,^7^ although this should not be construed to show that CRD has protective properties.^72^ According to our pooled studies, the CRD prevalence ranged from 2.1% to 24.0%.

A few studies have indicated that comorbid asthma is not associated with severe COVID‐19.^21^, ^22^, ^43^, ^47^, ^55^, ^59^, ^68^ Only two studies reported a significantly higher prevalence of comorbid asthma in patients with severe COVID‐19.^19^, ^53^ Several other studies have reported an increased prevalence of comorbid asthma in patients with severe COVID‐19, although no statistical data were provided. One study conducted by Grein et al.^35^ reported a 14.7% prevalence of comorbid asthma in patients with severe COVID‐19 and a 5.2% prevalence of asthma in patients with non‐severe COVID‐19, although no further statistical data were provided. Our results related to comorbid asthma constitute one of the most important findings in our meta‐analysis, indicating that this condition is not associated with severe COVID‐19. Naturally, this topic requires further research.

Viral infections affecting the respiratory system can provoke asthma attacks and COPD exacerbations by increasing the immune response and inflammation.^73^ The use of inhaled glucocorticosteroids (ICS) in patients with COPD and asthma is known to produce undesirable side effects, including an increased risk of pneumonia and upper respiratory tract infections.^74^, ^75^, ^76^ It is important to note, however, that these two different groups of patients may demonstrate different immune responses to these types of infections. A recent study has reported that ciclesonide, an ICS, can reduce the cytopathic activity of SARS‐CoV‐2 and may be useful in preventing COVID‐19 and reducing its severity.^77^ In our study, we found that patients with asthma did not have a higher risk of severe COVID‐19. As some of these patients were likely to have a history of allergy/atopy, ICSs may have been prescribed more frequently to this patient population when compared to those with CRD and COPD. Moving forward, more detailed studies are needed to investigate the relationship between COVID‐19, asthma and inhaled steroids.

In the coming months, potential vaccinations for COVID‐19 will be an important focus of attention. Although case fatality rates in different countries vary considerably, it has been demonstrated that many factors (comorbid diseases, age, etc.) can affect mortality rates and that patients with COPD have an increased risk of a more severe disease course and higher mortality. Vaccination studies for SARS‐CoV‐2 are ongoing and likely to produce further results in the coming months. If these are successful, prioritizing patients at risk of severe COVID‐19, like those with COPD, for vaccination is very important.

### Interstitial lung diseases and COVID‐19

An increase in the severe course of COVID‐19 was not reported in a retrospective study of 401 interstitial lung disease (ILD) patients receiving immunosuppressive therapy.^78^ Contrary to this finding, patients with ILD in another study had an increased mortality (HR = 1.60, 95% CI = 1.17–2.18, *p* = 0.003) due to COVID‐19, especially those with obesity and poor respiratory function parameters, and the highest mortality has been reported in rheumatoid ILD and chronic hypersensitivity pneumonitis.^79^ Huang et al.^80^ reported that higher d‐dimer and IL‐1β, IL‐8 and IL‐10 levels were observed in COVID‐19 patients with ILD than in COVID‐19 patients without ILD. However, whether the immunosuppressive therapies used by ILD patients predispose to greater susceptibility to COVID‐19 and infection by other opportunistic pathogens is not yet clear. Whether immunosuppressive therapies prevent the abnormal inflammatory response and cytokine storm that may develop during the course of severe COVID‐19 is also subject to future research.

### Cystic fibrosis and COVID‐19

Limited information is available on cystic fibrosis (CF) and COVID‐19. The most detailed information on this subject has come from a project carried out by the European Cystic Fibrosis Society (COVID‐CF project in Europe). According to this project, 268 cases of CF and COVID‐19 have been reported, of which 12 patients (4.5%) needed ICU care and five patients (1.9%) died.^81^ The lower‐than‐expected prevalence and mortality of COVID‐19 in patients with CF can be explained by the increased attention of patients to personal protection due to their illness. In addition, the potentially protective role of additional long‐term treatments (such as DNase and azithromycin/tobramycin) that these patients receive requires further research.^82^

### Bronchiectasis and COVID‐19

Bronchiectasis, similar to other rare lung diseases, is also under‐reported in studies. Some of the patients defined as having CRD, chronic pulmonary disease or lung disease in included studies may be patients with bronchiectasis. Newly defined cases of bronchiectasis after severe COVID‐19 infection are also increasing.^83^

In the present meta‐analysis, a total of 53 articles were reviewed with the largest sample size of all previous related studies (*n* = 658,073); however, this meta‐analysis contained some unavoidable limitations. Nearly half of the data were generally of Chinese origin and included only English articles that were published between January and October 2020. Even though the vast majority of included studies classified patients as having severe versus non‐severe disease,^13^, ^23^, ^24^, ^29^, ^32^, ^36^, ^38^, ^41^, ^47^, ^48^, ^52^, ^56^, ^60^, ^62^, ^64^, ^65^, ^67^, ^69^ some studies used different terminologies, including survivors/non‐survivors,^21^, ^22^, ^25^, ^26^, ^28^, ^33^, ^37^, ^39^, ^40^, ^44^, ^50^, ^51^, ^53^, ^54^, ^55^, ^58^, ^61^, ^63^, ^70^ need for ICU or IMV/non‐ICU or non‐IMV,^19^, ^20^, ^27^, ^34^, ^35^, ^43^, ^45^, ^57^, ^59^, ^68^ good/poor outcomes,^30^ general treatment/refractory to treatment^49^ and improvement/progression.^46^, ^66^ Although the terminology differed among studies, the characteristics of more severe cases (need for ICU/IMV, critical, non‐survivors, progression and refractory) seemed to comply with the general framework of our study.

Furthermore, all studies lack information regarding the definition of comorbid COPD, asthma and CRD. Only nine of the studies were prospective,^21^, ^24^, ^26^, ^30^, ^33^, ^35^, ^37^, ^42^, ^46^ whereas the rest of the included studies were planned retrospectively. Patient follow‐up times are also very limited in all studies. We analysed patients with recent disease and short‐term follow‐up; however, differences may become more evident during long‐term follow‐up. In addition, many studies are published every day. The increase in pre‐print publication (without evaluation by a reviewing process) style makes it more difficult to stay up to date than before. Our meta‐analysis has not yet adequately addressed the question of the potential impact of greater age of patients with COPD compared to the average patients with asthma, and this should be an important component for future analysis. Finally, we limited our meta‐analysis to studies including patients with COPD, asthma and CRD because current research has not yet explored the relationship of COVID‐19 with other chronic lung diseases, including bronchiectasis, CF, ILDs and sarcoidosis. Further studies should investigate these relationships in more detail.

In conclusion, comorbid COPD and CRD were clearly associated with higher severity of COVID‐19; however, no association between asthma and severe COVID‐19 was identified. Questions remain regarding the relationships between COVID‐19 and the severity of COPD and asthma, as well as the relationship of COVID‐19 with other pulmonary conditions, including ILDs, bronchiectasis and CF.

## AUTHOR CONTRIBUTIONS

**Inke R. König:** Methodology; supervision; validation; writing‐review & editing. **Uta Jappe:** Funding acquisition; investigation; supervision; writing‐review & editing. **Daniel Drömann:** Data curation; resources; writing‐review & editing. **Askin Gulsen:** Conceptualization; data curation; formal analysis; methodology; project administration; resources; software; visualization; writing‐original draft; writing‐review & editing.

## CONFLICT OF INTEREST

Uta Jappe reports grants and personal fees from the Federal Ministry of Education and Research, German Center for Lung Research (DZL), during the conduct of the study. Inke R. König reports grants from German Research Foundation and Federal Ministry of Education and Research, outside the submitted work. The other authors declare that they have no conflicts of interest.

## Supporting information


**Figure S1.** Funnel plots with pseudo 95% confidence limits.
**Table S1.** Systematic search strategy.
**Table S2.** Prevalence of COPD in patients with severe versus non‐severe COVID‐19.
**Table S3.** Prevalence of CRD in patients with severe versus non‐severe COVID‐19.
**Table S4.** Prevalence of asthma in patients with severe versus non‐severe COVID‐19.

**Visual Abstract** Effect of comorbid pulmonary disease on severity of COVID‐19: A systematic review & meta‐analysis.

## Data Availability

The data sets analysed during the current study are available from the corresponding author on reasonable request. Trial Registration: CRD42020179122 at PROSPERO https://www.crd.york.ac.uk/prospero
