# Supplementary material for: Effect of comorbid pulmonary disease on the severity of COVID‐19: A systematic review and meta‐analysis
Source: Respirology. 2021 May 6;26(6):552–65. doi: 10.1111/resp.14049 (PMC8207055; doi:10.1111/resp.14049)
Supplement: Supplementary file 1 — Figure S1. Funnel plots with pseudo 95% confidence limits. Table S1. Systematic search strategy. Table S2. Prevalence of COPD in patients with severe versus non‐severe COVID‐19. Table S3. Prevalence of CRD in patients with severe versus non‐severe COVID‐19. Table S4. Prevalence of asthma in patients with severe versus non‐severe COVID‐19. [file RESP-26-552-s002.docx]

**SUPPORTING INFORMATION**

**Effect of comorbid pulmonary disease on severity of COVID-19: a systematic review and meta-analysis**

**Askin Gülsen^1^, Inke R. König^2^, Uta Jappe^1^, Daniel Drömann^3^**

^1^ Division of Clinical and Molecular Allergology, Research Center Borstel, Leibniz Lung Center, Airway Research Center North (ARCN), Member of the German Center for Lung Research (DZL), Borstel, Germany; Interdisciplinary Allergy Outpatient Clinic, Department of Pneumology, University of Luebeck, Germany
^2^ Institute of Medical Biometry and Statistics, University of Luebeck, Germany, Airway Research Center North (ARCN), Member of the German Center for Lung Research (DZL), University of Luebeck, Germany
^3^ Department of Pneumology, Airway Research Center North (ARCN), Member of the German Center for Lung Research (DZL), University of Luebeck, Germany

**Figure S1***.* Funnel plots with pseudo 95% confidence limits


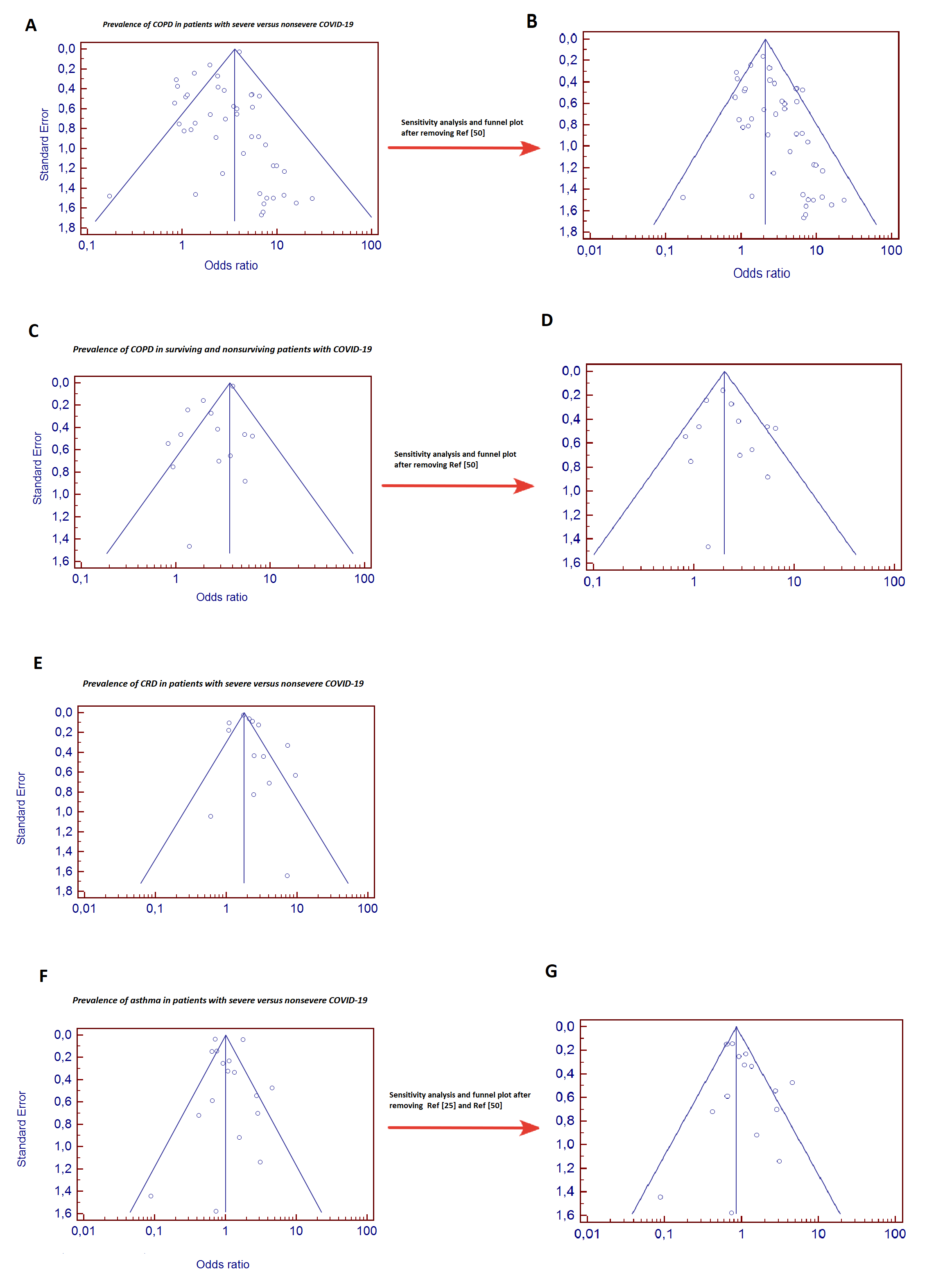


**Table S1-** **Systematic search strategy**

| **Line #** | **Mesh terms and text words** |
| --- | --- |
| **#1** | (COVID-19) OR (2019-nCoV) OR (SARS-CoV-2) OR (novel coronavirus) |
| **#2** | #1 AND (Asthma) |
| **#3** | #1 AND (COPD) |
| **#4** | #1 AND (Respiratory Disease) |
| **#5** | #2, OR #3, OR #4 AND (clinical characteristics) |
| **#6** | #2, OR #3, OR #4 AND (risk factor) |

| **Table S2. Prevalence of COPD in patients with severe versus non-severe COVID-19** | | | | | | | | | |
| --- | --- | --- | --- | --- | --- | --- | --- | --- | --- |
|  | **Severe COVID-19 patients with COPD** | | | **Non-severe COVID-19 patients with COPD** | | | | **Results** | |
| **First Author** | **Event** | **Total** | **%** | **Event** | **Total** | | **%** | **OR** | ***P**** |
| **Almazeedi S. ^[19]^** | 2 | 42 | 4.8 | 37 | 1,054 | | 3.5 | 1.37 | - |
| **Argenziano MG. ^[20]^** | 14 | 236 | 5.9 | 52 | 764 | | 6.8 | 0.86 | - |
| **Auld SC. ^[21]^** | 5 | 62 | 8.1 | 14 | 147 | | 9.5 | 0.83 | 0.737 |
| **Buckner FS. ^[23]^** | 7 | 51 | 13.7 | 4 | 54 | | 7.4 | 1.98 | - |
| **Caratozzolo S. ^[26]^** | 8 | 41 | 19.5 | 65 | 807 | | 8.0 | 2.76 | 0.019 |
| **Chen Q. ^[13]^** | 0 | 43 | 0 | 6 | 102 | | 5.9 | 0.17 | 0.18 |
| **Cai Q. ^[24]^** | 13 | 91 | 14.3 | 19 | 292 | | 6.5 | 2.39 | 0.03 |
| **Feng X. ^[30]^** | 2 | 20 | 10.0 | 9 | 94 | | 9.5 | 1.04 | >0.99 |
| **Feng Y. ^[31]^** | 14 | 124 | 11.3 | 8 | 352 | | 2.3 | 5.47 | <0.001 |
| **Gao Y. ^[32]^** | 3 | 15 | 20.0 | 0 | 28 | | 0 | 15.9 | 0.037 |
| **Giorgi Rossi P. ^[33]^** | 24 | 217 | 11.0 | 91 | 1,075 | | 8.5 | 1.34 | - |
| **Goyal P. ^[34]^** | 7 | 130 | 5.3 | 13 | 263 | | 4.9 | 1.09 | - |
| **Guan WJ. ^[36]^** | 6 | 173 | 3.4 | 6 | 926 | | 0.6 | 5.50 | - |
| **Gupta S. ^[37]^** | 87 | 784 | 11.1 | 86 | 1,431 | | 6.0 | 1.95 | - |
| **Güner R. ^[38]^** | 6 | 50 | 12.0 | 6 | 172 | | 3.5 | 3.77 | 0.019 |
| **He Y. ^[40]^** | 22 | 133 | 16.5 | 6 | 203 | | 3.0 | 6.50 | <0.001 |
| **Hu L. ^[41]^** | 6 | 172 | 3.5 | 0 | 151 | | 0 | 11.8 | 0.033 |
| **Huang C. ^[42]^** | 1 | 13 | 7.6 | 0 | 28 | | 0 | 6.84 | 0.14 |
| **Israelsen SB. ^[43]^** | 2 | 27 | 7.4 | 9 | 148 | | 6.1 | 1.23 | 1.00 |
| **Javanian M. ^[44]^** | 5 | 19 | 26.3 | 7 | 81 | | 8.6 | 3.77 | 0.032 |
| **Lagi F. ^[45]^** | 3 | 16 | 18.7 | 2 | 68 | | 2.9 | 7.61 | 0.045 |
| **Liu W. ^[46]^** | 1 | 11 | 9.1 | 1 | 67 | | 1.5 | 6.60 | 0.264 |
| **Li X. ^[47]^** | 13 | 269 | 4.8 | 4 | 279 | | 1.4 | 3.49 | 0.26 |
| **Li YK. ^[48]^** | 4 | 9 | 44.4 | 1 | 16 | | 6.2 | 12.0 | 0.038 |
| **Mo P. ^[49]^** | 4 | 85 | 4.7 | 0 | 70 | | 0 | 7.78 | 0.489 |
| **Parra-Bracamonte GM. ^[50]^** | 1,839 | 38,310 | 4.7 | 3,619 | 292,988 | | 1.2 | 4.03 | - |
| **Parantje I. ^[51]^** | 28 | 310 | 9.0 | 31 | 768 | | 4.0 | 2.36 | - |
| **Salacup G. ^[53]^** | 7 | 52 | 13.5 | 23 | 190 | | 12.1 | 1.12 | 0.813 |
| **Shi S. ^[54]^** | 2 | 62 | 3.2 | 21 | 609 | | 3.4 | 0.93 | 1.000 |
| **Tomlins S. ^[55]^** | 4 | 20 | 20.0 | 6 | 75 | | 8.0 | 2.87 | 0.20 |
| **Wan S. ^[56]^** | 4 | 40 | 10.0 | 0 | 95 | | 0 | 23.5 | -- |
| **Wang D. ^[57]^** | 3 | 36 | 8.3 | 1 | 102 | | 0.9 | 9.18 | 0.054 |
| **Wang L. ^[58]^** | 11 | 65 | 16.9 | 10 | 274 | | 3.6 | 5.37 | <0.001 |
| **Wang Z. ^[59]^** | 2 | 14 | 14.2 | 2 | 55 | | 3.6 | 4.41 | 0.181 |
| **Wu J. ^[60]^** | 1 | 83 | 1.2 | 0 | 197 | | 0 | 7.18 | - |
| **Yan X. ^[61]^** | 0 | 40 | 0 | 8 | 964 | | 8.3 | 1.38 | 0.563 |
| **Yang P. ^[62]^** | 4 | 68 | 5.8 | 0 | 65 | | 0 | 9.14 | 0.047 |
| **Zhang JJ. ^[64]^** | 2 | 58 | 3.4 | 0 | 82 | | 0 | 7.30 | 0.170 |
| **Zhang G. ^[65]^** | 4 | 55 | 7.2 | 2 | 166 | | 1.2 | 6.43 | 0.035 |
| **Zhang J ^[66]^** | 1 | 18 | 5.6 | 2 | 93 | | 2.2 | 2.67 | 0.415 |
| **Zhang R. ^[67]^** | 3 | 30 | 10.0 | 1 | 90 | | 1.1 | 9.88 | 0.048 |
| **Zhao Z. ^[68]^** | 11 | 195 | 5.6 | 25 | 398 | | 6.3 | 0.89 | 0.76 |
| **Zheng F. ^[69]^** | 2 | 30 | 6.7 | 4 | 131 | | 3.1 | 2.26 | 0.346 |
| **Zhou F. ^[70]^** | 4 | 54 | 7.4 | 2 | 137 | | 1.4 | 5.40 | 0.047 |
|  | | | | | | | | | |
| **Total (random)** | 2,191 | 42,373 | 5.2% | 4,203 | 306,151 | 1.4% | | 2.58 | p<0.001 |
| **Total (fixed)** |  |  |  |  |  |  | | 3.61 | p<0.001 |
| Test for heterogeneity: Tau^2^: Q=131.5, DF=43 (p<0.001); I^2^=67.31%  **Test for overall effect:** z=7.15 (p<0.001)  *The p values given in the table were taken from the studies. W, weight; OR, odds ratio; CI, confidence interval. | | | | | | | | | |

| **Table S3. Prevalence of CRD in patients with severe versus non-severe COVID-19** | | | | | | | | |
| --- | --- | --- | --- | --- | --- | --- | --- | --- |
|  | **Severe COVID-19 patients with CRD** | | | **Non-severe COVID-19 patients with CRD** | | | **Results** | |
| **First Author** | **Event** | **Total** | **%** | **Event** | **Total** | **%** | **OR** | ***P**** |
| **Argenziano MG. ^[20]^** | 55 | 236 | 23.3 | 168 | 764 | 22.0 |  | - |
| **Berenguer J. ^[22]^** | 310 | 1,116 | 28.8 | 405 | 2,879 | 14.1 |  | <0.001 |
| **Cao J. ^[25]^** | 4 | 17 | 23.5 | 6 | 85 | 7.1 | 4.05 | 0.101 |
| **CDC report ^[27]^** | 94 | 457 | 20.5 | 515 | 6,180 | 8.3 | 2.85 | - |
| **Deng Y. ^[28]^** | 22 | 109 | 20.1 | 3 | 116 | 2.5 | 9.52 | <0.001 |
| **ECDC, Week42 ^[29]^** | 2,649 | 39,609 | 6.7 | 9,350 | 237,188 | 3.9 |  | - |
| **Gupta S. ^[37]^** | 197 | 784 | 25.1 | 334 | 1,431 | 23.3 |  | - |
| **Harrison SL. ^[39]^** | 391 | 1,296 | 30.1 | 5,122 | 30,165 | 17.0 |  | - |
| **Hu L. ^[41]^** | 21 | 172 | 12.2 | 8 | 151 | 5.3 |  | 0.010 |
| **Qi D. ^[52]^** | 10 | 50 | 20.0 | 15 | 217 | 6.9 | 3.37 | 0.009 |
| **Wan S. ^[56]^** | 1 | 40 | 2.5 | 0 | 95 | 0 | 7.25 | - |
| **Wu J. ^[60]^** | 3 | 83 | 3.6 | 3 | 197 | 1.5 | 2.42 | - |
| **Yan X. ^[61]^** | 21 | 40 | 52.5 | 126 | 964 | 13.1 |  | <0.001 |
| **Yang X. ^[63]^** | 2 | 32 | 6.2 | 2 | 20 | 10 | 0.60 | - |
|  | | | | | | | | |
| **Total (random)** | 3,780 | 44,041 | 8.6% | 16,057 | 280,447 | 5.7% | 2.14 | p<0.001 |
| **Total (fixed)** |  |  |  |  |  |  | 1.80 | p<0.001 |
| Test for heterogeneity: Q=93.2, DF=13 (p<0.001); I^2^=86.0%  **Test for overall effect**: z=7.1 (p<0.001)  *The p values given in the table were obtained from the studies.  CRD, chronic respiratory diseases; W, weight; OR, odds ratio; CI, confidence interval. | | | | | | | | |

| **Table S4. Prevalence of asthma in patients with severe versus non-severe COVID-19** | | | | | | | | |
| --- | --- | --- | --- | --- | --- | --- | --- | --- |
|  | **Severe COVID-19 patients with Asthma** | | | **Non-severe COVID-19 patients with Asthma** | | | **Results** | |
| **First Author** | **Event** | **Total** | **%** | **Event** | **Total** | **%** | **OR** | ***P**** |
| **Almazeedi S. ^[19]^** | 6 | 42 | 14.3 | 37 | 1,054 | 3.5 | 4.58 | <0.0001 |
| **Argenziano MG. ^[20]^** | 29 | 236 | 12.3 | 84 | 764 | 11.0 | 1.13 | - |
| **Auld SC. ^[21]^** | 4 | 62 | 6.4 | 14 | 147 | 9.5 | 0.65 | 0.470 |
| **Berenguer J. ^[22]^** | 69 | 1,116 | 6.2 | 230 | 2,884 | 8.0 | 0.76 | 0.053 |
| **Buckner FS. ^[23]^** | 3 | 51 | 5.9 | 7 | 54 | 13.0 | 0.42 | - |
| **ECDC, Week42 ^[29]^** | 841 | 39,609 | 2.1 | 2,869 | 237,188 | 12.1 | 1.77 | - |
| **Goyal P. ^[34]^** | 17 | 130 | 13.0 | 32 | 263 | 12.1 | 1.08 | - |
| **Grein J. ^[35]^** | 5 | 34 | 14.7 | 1 | 19 | 5.2 | 3.10 | - |
| **Gupta S. ^[37]^** | 70 | 784 | 8.9 | 188 | 1,431 | 13.1 | 0.64 | - |
| **Israelsen SB. ^[43]^** | 6 | 27 | 22.2 | 14 | 148 | 9.4 | 2.73 | 0.11 |
| **Li X. ^[47]^** | 3 | 269 | 1.1 | 2 | 279 | 0.7 | 1.56 | 0.681 |
| **Parra-Bracamonte GM. ^[50]^** | 777 | 38,310 | 2.0 | 8,206 | 292,988 | 2.8 | 0.71 | - |
| **Parantje I. ^[51]^** | 23 | 310 | 7.4 | 61 | 768 | 7.9 | 0.92 | - |
| **Salacup G. ^[53]^** | 0 | 52 | 0 | 18 | 190 | 9.5 | 0.08 | 0.021 |
| **Tomlins S. ^[55]^** | 4 | 20 | 20.0 | 6 | 75 | 8.0 | 2.87 | >0.9 |
| **Wang Z. ^[59]^** | 0 | 14 | 0 | 2 | 55 | 3.6 | 0.73 | 0.633 |
| **Zhang JJ. ^[64]^** | 0 | 58 | 0 | 0 | 82 | 0 | 1.41 | - |
| **Zhao Z. ^[68]^** | 16 | 195 | 8.2 | 25 | 398 | 6.3 | 1.33 | 0.76 |
|  |  |  |  |  |  |  |  |  |
| **Total (random)** | 1,873 | 81,319 | 2.3% | 11,796 | 538,787 | 2.2% | 1.13 | 0.50 |
| **Total (fixed)** |  |  |  |  |  |  | 1.01 | 0.59 |
| Test for heterogeneity: Q=317.1, DF=16 (p<0.001); I^2^=94.5%  Test for overall effect: z=-0.66 (p=0.50)  *The p values given in the table were obtained from the studies.  W, weight; OR, odds ratio; CI, confidence interval. | | | | | | | | |
