# Supplementary material for: Effect of comorbid pulmonary disease on the severity of COVID‐19: A systematic review and meta‐analysis
Source: Respirology. 2021 May 6;26(6):552–65. doi: 10.1111/resp.14049 (PMC8207055; doi:10.1111/resp.14049)
Supplement: Supplementary file 2 — Visual Abstract Effect of comorbid pulmonary disease on severity of COVID‐19: A systematic review & meta‐analysis. [file RESP-26-552-s001.pdf]

# Effect of comorbid pulmonary disease on severity of COVID-19:
## A systematic review & meta-analysis

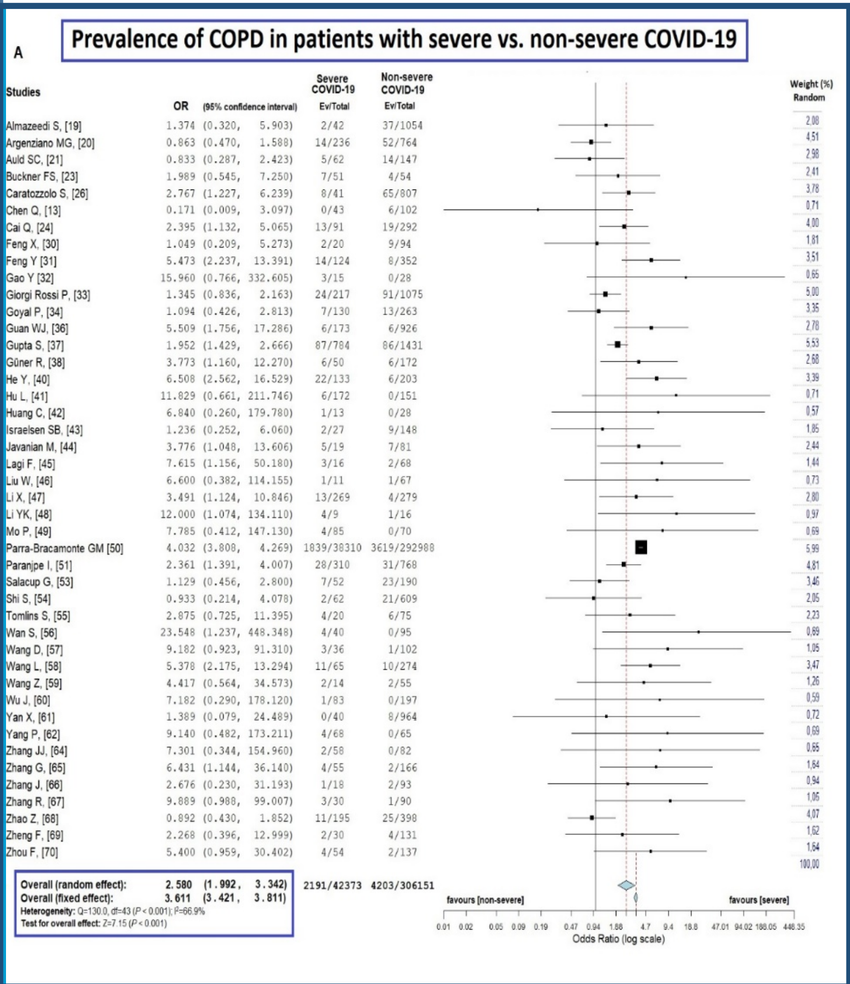


## Summary Points

Comorbid COPD and chronic respiratory diseases (CRD) were clearly associated with higher severity of COVID-19; however, no association between asthma and severe COVID-19 was identified.

*

Questions remain regarding the relationships between COVID-19 and the severity of COPD and asthma, as well as the relationship of COVID-19 with other pulmonary conditions, including interstitial lung diseases, bronchiectasis, and cystic fibrosis.

*

The potential impact of greater age of patients with COPD compared to the average patients with asthma should be an important component for future analysis.

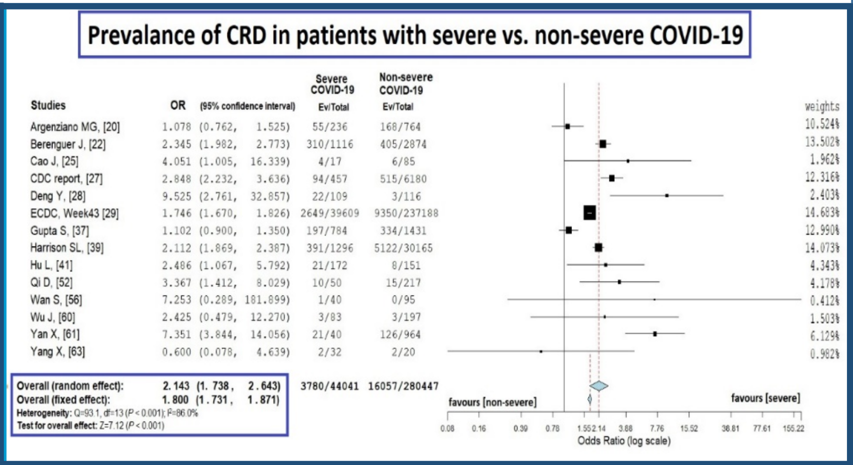


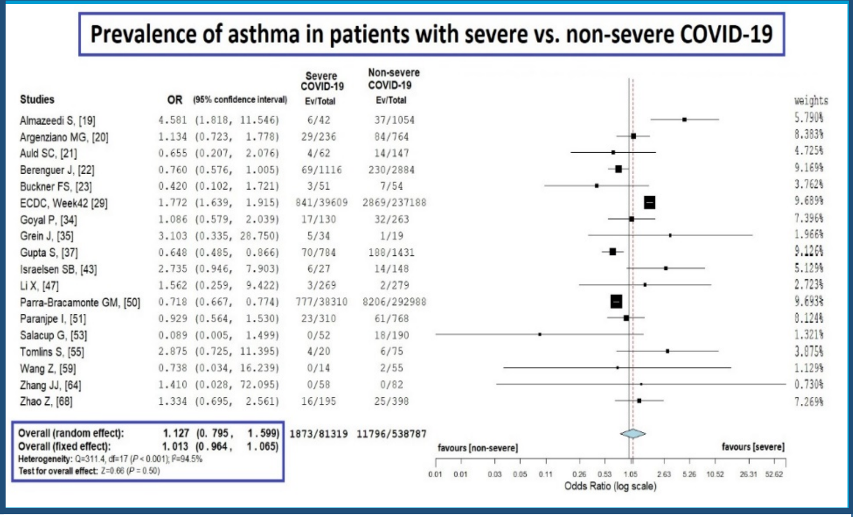


Respirology

*Effect of comorbid pulmonary disease on severity of COVID-19: A systematic review and meta-analysis*

Gülsen et al. Respirology 2021; DOI: 10.1111/resp.14049
